# Immunological Pre‐Metastatic Niche in Dogs With Naturally Occurring Osteosarcoma

**DOI:** 10.1111/vco.13026

**Published:** 2024-11-11

**Authors:** Mikael Kerboeuf, Kristin Paaske Anfinsen, Erling Olaf Koppang, Frode Lingaas, David Argyle, Jon Teige, Bente Kristin Sævik, Lars Moe

**Affiliations:** ^1^ Department of Preclinical Sciences and Pathology, Faculty of Veterinary Medicine Norwegian University of Life Sciences Ås Norway; ^2^ Department of Companion Animal Clinical Sciences, Faculty of Veterinary Medicine Norwegian University of Life Sciences Ås Norway; ^3^ The Royal (Dick) School of Veterinary Studies and Roslin Institute University of Edinburgh Midlothian UK; ^4^ AniCura Jeløy Small Animal Hospital Moss Norway

**Keywords:** bone cancer, canine, metastasis, myeloid cells, osteosarcoma

## Abstract

Pre‐metastatic niche (PMN) formation is essential for metastatic development and drives organotropism. Tumour‐derived extracellular vesicles and soluble factors remodel the microenvironment of distant metastatic organs before subsequent metastasis. Dogs with osteosarcoma (OS) have proven to be excellent disease models for their human companions. Here, we show evidence of PMN formation in dogs with OS before metastasis. We necropsied and sampled lung tissues from dogs with naturally occurring treatment‐naïve OS (*n* = 15) and control dogs without cancer (*n* = 10). We further divided dogs with OS into those having lung metastases (*n* = 5) and those without (*n* = 10). We stained formalin‐fixed paraffin‐embedded tissues using multiplex immunofluorescence to quantify the number of bone marrow‐derived cells, monocytes and macrophages in the lung samples from each dog. The numbers of CD204^+^ macrophages, CD206^+^ macrophages and monocytes and CD11d^+^ bone marrow‐derived cells (BMDCs) were significantly higher in the pre‐metastatic lung of dogs with OS (*n* = 10) than in control dogs without cancer (*n* = 10). Furthermore, the total nucleated cell (DAPI^+^) density was higher before metastasis than in healthy lungs. In dogs with established metastases, the number of CD11d^+^ BMDCs was significantly lower than in the pre‐metastatic lung, suggesting this recruitment is transient. Our study provides evidence of PMN existence in a naturally occurring cancer model similar to those observed in pre‐clinical murine models. BMDCs are recruited to the lungs before metastases have developed. Dogs with OS may represent ideal candidates for assessing new PMN‐targeting therapies.

## Graphical abstract

Schematic representation of the study. Dogs with naturally occurring osteosarcoma (OS) were used as models to study pre‐metastatic niche formation. Dogs were necropsied, and lung biopsies were collected from dogs with OS before metastasis had occurred. Dogs with established OS metastases and dogs without cancer were used as controls. There were significantly higher numbers of CD204^+^ and CD206^+^ macrophages, CD206^+^ monocytes and CD11d^+^ bone marrow‐derived cells (BMDCs) in the lungs of dogs with OS before metastasis than in controls without cancer. Furthermore, dogs with OS with established metastases had fewer CD11d+ BMDCs than those without.

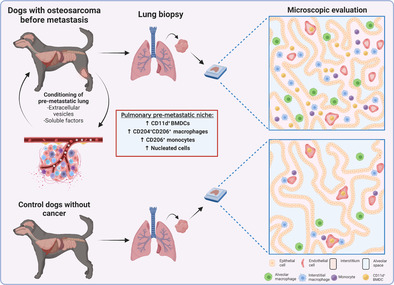

## 1 Introduction

Metastasis is the primary cause of death among cancer patients [1]. Different cancers exhibit specific metastatic patterns, a phenomenon first described by Dr. Steven Paget in his ‘Seed and Soil Hypothesis’ [2]. Over a century later, the concept of a pre‐metastatic niche (PMN) was introduced by Kaplan et al., offering an underlying mechanism for the apparent organotropism of metastasis [3]. Since then, PMN formation has been described in several cancers and different metastatic target organs in murine models [4, 5, 6]. The PMN is defined as the metastasis‐supporting microenvironmental changes that occur in a distant target organ before metastasis [6]. These microenvironmental changes include angiogenesis and vascular permeability, lymphangiogenesis, stromal and metabolic reprogramming, inflammation and immunosuppression [6]. The primary tumour releases soluble factors and extracellular vesicles (EVs) containing proteins, lipids, mRNA, miRNA and DNA that remodel the microenvironment at the metastatic sites [4, 6]. In murine models of melanoma and breast cancer, tumour‐derived EVs show distinctive organotropism and induce PMN‐formation in organs that subsequently develop metastasis [7, 8, 9]. Metastasis can, to some extent, be blocked when mice are pre‐treated with EVs from non‐metastatic melanoma and breast cancer lines before tumour transplantation [7, 8, 9]. The lungs are a common predilection site for metastasis and have been the focus of many PMN studies. In murine models, the immunological changes here are attributed mainly to the recruitment of bone marrow‐derived cells (BMDCs) [4, 6]. Increased numbers of CD11b^+^ BMDCs, myeloid‐derived suppressor cells (MDSCs), VEGFR1^+^ haematopoietic progenitor cells, monocytes, granulocytes, regulatory T‐cells (Tregs) and macrophages have been observed before metastasis in murine models of osteosarcoma (OS), breast cancer, melanoma, prostate cancer, salivary adenoid cystic carcinoma, non‐small cell lung carcinoma and pancreatic cancer [6]. In addition, NK‐cell dysfunction and a hampered cytotoxic T‐cell response have been demonstrated in murine models of breast cancer and pancreatic cancer. Despite findings in murine models, no studies have confirmed that distant PMN occurs in naturally occurring cancers in humans or animals apart from in tumour‐draining lymph nodes [10].

Dogs with naturally occurring OS are considered excellent models for their human counterparts [11, 12]. OS is the most common primary bone tumour in humans and dogs, with remarkably similar clinical, pathological, molecular and genetic features [12, 13]. Furthermore, treatment strategies are the same between the two species, and the disease is more prevalent in dogs than humans. Despite the aggressive nature of OS, with most dogs (> 90%) developing pulmonary metastasis after complete surgical excision of the primary tumour, only a small proportion of dogs have visible pulmonary metastases (< 15%) or micrometastasis (20% of those without visible metastases) at clinical presentation [14, 15, 16, 17].

Here, we show that dogs with naturally occurring OS exhibit similar immunological changes in the lungs before metastasis to those seen in murine PMN models. We provide evidence of PMN existence in a naturally occurring cancer model. The numbers of CD11d^+^ BMDCs, CD204^+^ macrophages and CD206^+^ macrophages and monocytes are higher in the pre‐metastatic lung of dogs with OS than in controls without cancer. Our results suggest that PMN formation occurs in dogs with OS and that they could serve as excellent models for further studies and as a bridge between pre‐clinical models and humans.

## 2 Materials and Methods

### 2.1 Study Population

The study was conducted as a prospective case series of dogs with OS (OS+) and retrospectively enrolled control dogs without OS (OS−). Dogs in the OS+ group could be of any breed, age and sex and had to have a histopathological diagnosis of OS at an appendicular site. The dogs in the OS− group were selected retrospectively from the pathology archives. They could be of any breed, age and sex and had to have been euthanised for another reason than cancer. Dogs were excluded from the study if they had any concurrent or previous neoplastic disease, concurrent systemic inflammatory disease or clinically detectable atopic or allergic disease. In addition, dogs were excluded if they had received chemotherapy, immunomodulating drugs, radiotherapy, immunotherapy or surgical treatment for their OS. All included cases were privately owned dogs, and owners had to sign a written consent form before euthanasia and necropsy.

### 2.2 Necropsy and Tissue Collection

All included dogs underwent standard necropsy after euthanasia. Organs were examined macroscopically, and routine tissue samples were collected (liver, kidneys, spleen, myocardium and lungs). In addition, tissue samples were collected from any suspected pathological lesions. Dogs in the OS+ group were divided into those having macroscopic metastases (OS+/Met+) and those without (OS+/Met−). Formalin‐fixed paraffin‐embedded (FFPE) tissues were sectioned, stained with haematoxylin & eosin (H&E) and examined by a single pathologist. Additional tissue samples were collected from the peripheral and central regions of each lung lobe from dogs in the OS+ groups. Sections from the dogs in the OS+/Met− group were previously immunohistochemically labelled with TP‐3 which recognises an isotype of alkaline phosphatase to identify micrometastases [17]. Micrometastases were identified in two of these dogs as summarised in Table 1. One of the peripheral lung samples was chosen using an online random number generator (www.random.org) for use in this study by the principal investigator. Only one lung tissue sample was available from the OS− dogs and used in this study, routinely taken from the peripheral areas.

**TABLE 1:** Overview of clinical characteristics and cause of euthanasia for dogs included in the study based on clinical records and necropsy findings.

Case	Group	Breed	Sex (M/F)	Age (years)	Year of sampling	Presence of pulmonary micrometastases (Y/N)	Cause of euthanasia
1	OS+/MET+	Mixed breed	M	1	2012	N.a.	Osteosarcoma, left distal radius
2	OS+/MET+	Giant Schnauzer	F	8	2013	N.a.	Osteosarcoma, right distal radius
3	OS+/MET+	Irish wolfhound	F	7	2014	N.a.	Osteosarcoma, right proximal humerus
4	OS+/MET+	Rottweiler	M	9	2013	N.a.	Osteosarcoma, left distal ulna
5	OS+/MET+	Tervuren	F	8	2014	N.a.	Osteosarcoma, left distal radius
6	OS+/MET−	Newfoundland dog	M	8	2018	No	Osteosarcoma, left distal radius
7	OS+/MET−	Siberian husky	M	3	2013	No	Osteosarcoma, left proximal humerus
8	OS+/MET−	Irish wolfhound	F	6	2013	Yes	Osteosarcoma, right distal tibia
9	OS+/MET−	English setter	M	8	2018	No	Osteosarcoma, right distal ulna
10	OS+/MET−	Pointer	F	4	2016	No	Osteosarcoma, left distal tibia
11	OS+/MET−	Shar Pei	M	11	2013	No	Osteosarcoma, right proximal humerus
12	OS+/MET−	Rottweiler	F	9	2015	Yes	Osteosarcoma, left proximal humerus
13	OS+/MET−	German shepherd	F	3	2013	No	Osteosarcoma, left distal radius
14	OS+/MET−	Flat‐coated retriever	M	3	2015	No	Osteosarcoma, left distal radius
15	OS+/MET−	Leonberger	M	6	2016	No	Osteosarcoma, left distal radius
16	OS−/MET−	Dalmatian	M	8	2016	N.a.	Urolithiasis
17	OS−/MET−	Shetland Sheepdog	F	1	2020	N.a.	Behavioural problems
18	OS−/MET−	Chihuahua	M	7	2017	N.a.	Idiopathic epilepsy
19	OS−/MET−	Jack Russell terrier	M	6	2018	N.a.	Road traffic accident (hip fracture, tail fracture, perforation jejunum and rectum, perforation bladder)
20	OS−/MET−	Australian shepherd	F	1	2019	N.a.	Behavioural problems
21	OS−/MET−	Dachshund	M	6	2014	N.a.	Intervertebral disc disease
22	OS−/MET−	Malinois	M	4	2016	N.a.	Intervertebral disc disease
23	OS−/MET−	Lagotto Romagnolo	M	1	2018	N.a.	Vertebral fracture
24	OS−/MET−	Spanish Greyhound	F	4	2015	N.a.	Behavioural problems
25	OS−/MET−	Dachshund	F	5	2014	N.a.	Road traffic accident (multiple hip fractures, fracture of sacral bone, subcutaneous, intramuscular and retroperitoneal bleeding)

### 2.3 Immunohistochemical Labelling

Tissues for immunohistochemical (IHC) labelling were sectioned from the FFPE blocks. Tissues were sectioned at 4 μm thickness using a microtome, mounted on poly‐lysin‐coated slides (SuperFrost Plus, Thermo Fisher Scientific, Oslo, Norway) and dried at room temperature for 1 h. The sections were stored at 4°C for up to 14 days until further staining. IHC labelling was performed using the peroxidase‐conjugated immune‐polymer method (EnVision+, Dako, Glostrup, Denmark). First, tissue sections were deparaffinised in xylene and rehydrated through an ethanol gradient using a standard protocol on an automated slide stainer. Next, heat‐induced epitope retrieval was performed in a pressure cooker at 110°C for 10 min using Diva Decloaked (Biocare Medical, Histolab, Gothenburg, Sweden). Endogenous peroxidase activity was inhibited by immersing the slides in a 3% H_2_O_2_ solution in methanol (4°C) for 10 min. The sections were blocked using a 1:50 solution of normal goat serum in 5% bovine serum albumin in tris‐buffered saline (BSA/TBS) for 30 min to prevent unspecific labelling. Sections were incubated with primary antibodies against CD204 (1:100, mouse anti‐human, IgG1, clone SRA‐E5, Abnova), CD206 (1:1600, rabbit anti‐human, IgG, polyclonal, Abcam) and CD11d (1:20, mouse anti‐dog, IgG1, clone CA18.3C6, Leukocyte Antigen Laboratory UC Davis), diluted in 1% BSA/TBS for 60 min. The samples were incubated with secondary antibodies, polymer‐HRP anti‐mouse or polymer‐HRP anti‐rabbit (EnVision+, Dako, Glostrup, Denmark), for 30 min. Immunolabelled tissues were developed using a 3‐amino‐9‐ethyl carbazole (AEC) substrate chromogen (EnVision+, Dako, Glostrup, Denmark) for 8–12 min and counterstained with Mayer's haematoxylin. The slides were mounted with coverslips using a water‐soluble mounting medium (Aquatex, Merck, Darmstadt, Germany) and left to dry at room temperature overnight. Negative controls were stained using the same protocol while omitting the primary antibodies. Washing between steps was done by immersing the slides in three changes of phosphate‐buffered saline (PBS) each for 5 min at room temperature. All incubations were done at room temperature in a moisture chamber on a rotation table. A section containing pulmonary metastases was used as a positive control for each staining and compared with the immunofluorescent staining (Figure S1).

### 2.4 Immunofluorescent Labelling

Tissues for immunofluorescent (IF) labelling were also sectioned from FFPE blocks. IF‐labelled tissues were compared to IHC‐labelled tissues to ensure similar labelling. Slides were sectioned, deparaffinised, and heat‐induced epitope retrieval was performed as described above. The slides were blocked using a 1:50 solution of normal goat serum in 5% BSA/TBS for 30 min. Sections were incubated with the same primary antibodies against CD204 (1:100), CD206 (1:1600) and CD11d (1:50), diluted in immunofluorescent buffer (IFB, PBS + 1% BSA + 2% foetal bovine serum, filtered thru a 0.2 μm filter) for 60 min at room temperature, in the dark. The samples were incubated with secondary antibodies (1:1000, Alexa fluor 647 goat anti‐mouse IgG1 and Alexa fluor 750 goat anti‐rabbit IgG, Thermo Fisher), diluted in IFB for 60 min at room temperature, in the dark. The three last washing steps were performed by adding DAPI to the wash buffer (1.43 μM, 1:10.000 dilution) for nuclear staining. The slides were mounted with #1.5 coverslips using ProLong Diamond Antifade mounting media (Thermo Fisher Scientific, Oslo, Norway) and placed at 4°C until image acquisition within 1 week. Washing between steps was performed using PBS + 0.05% Tween 20. Negative controls were stained using the same protocol while omitting the primary antibodies. As part of the protocol optimisation, fluorescent minus one (FMO) controls were labelled by excluding one of the primary antibodies or DAPI to ensure there was no unspecific binding of the secondary antibodies or spectral overlap between fluorophores (Figure S2). Single controls were labelled with only one of the primary antibodies or DAPI. In addition, single controls were labelled with one or both secondary antibodies to ensure there was no cross‐reactivity between primary and secondary antibodies. Isotype controls were also labelled to ensure there was no unspecific labelling by primary antibodies. A positive control from healthy splenic tissue was labelled for each staining.

### 2.5 Microscopic and Confocal Evaluation

IHC labelled slides were evaluated and imaged using a Zeiss AX10 microscope, equipped with a Zeiss axiocam 506 colour camera coupled with Zen pro‐2012 (blue edition) image‐acquiring software (Carl Zeiss Microscopy GmbH, Jena, Germany). IF‐labelled slides were imaged by a single evaluator using a Leica Stellaris 8 confocal microscope (Leica Microsystems, Wetzlar, Germany). Ten randomly selected high‐power field images (HPF, 200×, equivalent to 0.276 mm^2^) were acquired from each IF‐labelled slide, and immunolabelled cells were automatically quantified using ImageJ 1.51 K by a single‐blinded evaluator (National Institute of Health, USA). Briefly, images were opened in ImageJ, converted to 8‐bit grayscale, thresholded, inverted and cells analysed using the particle analysis tool (Figure S3). The number of CD204^+^, CD206^+^, CD11d^+^, CD204^−^CD206^+^, CD204^+^CD206^−^ and CD204^+^CD206^+^ cells were recorded and normalised against the total number of nuclei in each field to account for variation in tissue density. For the DAPI channel, the fluorophore was excited using the 405 nm laser, and the detector was set to record between 410 and 480 nm. For Alexa fluor 647, the fluorophore was excited by setting the white laser to 645 nm, while the detector was set to record between 650 and 700 nm. In addition, Tau Gating was set to record between 0.5 and 5 ns to remove unwanted autofluorescence. For Alexa fluor 750, the white laser was set to 685 nm, and the detector was set to record between 750 and 800 nm.

### 2.6 Statistical Analysis

All statistical analyses were performed using JMP pro 15.1.0 (SAS Institute Inc. Cary, NC). The mean number of CD204^+^, CD206^+^, CD204^+^CD206^−^, CD204^+^CD206^+^, CD204^−^CD206^+^, CD11d^+^ and DAPI positive cells per 10 HPF, as well as the number of positive cells normalised to the total nucleated cell count (DAPI positive), was compared between groups using Wilcoxon rank‐sum tests for each pair and an unpaired Kruskal‐Wallis test. The mean age and weight were compared between groups using Student t‐tests, while sex distribution was compared using a Chi‐square test. *p* values < 0.05 were considered statistically significant for statistical testing.

## 3 Results

### 3.1 Clinical Data

A total of 25 dogs were included in the study, euthanized and necropsied between 2012 and 2022. Fifteen cases were included in the OS+ group, five had macroscopic metastases (OS+/Met+) (Figure S4b,d) and ten did not (OS+/Met−) (Figure S4a,c). Ten dogs were included in the control group (OS−/Met−). An overview of clinical characteristics, cause of euthanasia and time of sampling are summarised in Table 1. The mean age of the OS+/Met+, OS+/Met− and OS−/Met− groups was 6.6 years (CI 2.6–10.6 years), 6.1 years (CI 4.1–8.1 years) and 4.3 years (CI 2.5–6.1 years) respectively. There were four (40%) females and six males (60%) in each of the OS+/Met− and OS−/Met− groups, and three females (60%) and two males (40%) in the OS+/Met+ group. There were no significant differences in age between the OS−/Met− and the OS+/Met− (*p* = 0.17) or the OS+/Met+ group (*p* = 0.15), nor between the OS+/Met− and OS+/Met+ group (*p* = 0.75). Similarly, there was no significant difference in sex distribution between the groups (*p* = 0.72). The mean weight of dogs in the OS−/Met− group (13.2 kg, CI 7.5–19.0) was significantly lower than for the dogs in the OS+/Met− group (39.8 kg, CI 26.8–50.7) and the OS+/Met+ group (39.5 kg, CI 18.8–60.2) (*p* = 0.0003 and *p* = 0.003, respectively). There was no significant difference between the mean weigh of dogs in the OS+/Met− and OS+/Met+ groups (*p* = 0.92).

### 3.2 Cases With OS Have Significantly Higher Numbers of CD204 ^+^ and CD206 ^+^ Macrophages in the Lungs Before Metastases Than Controls

**FIGURE 1:**
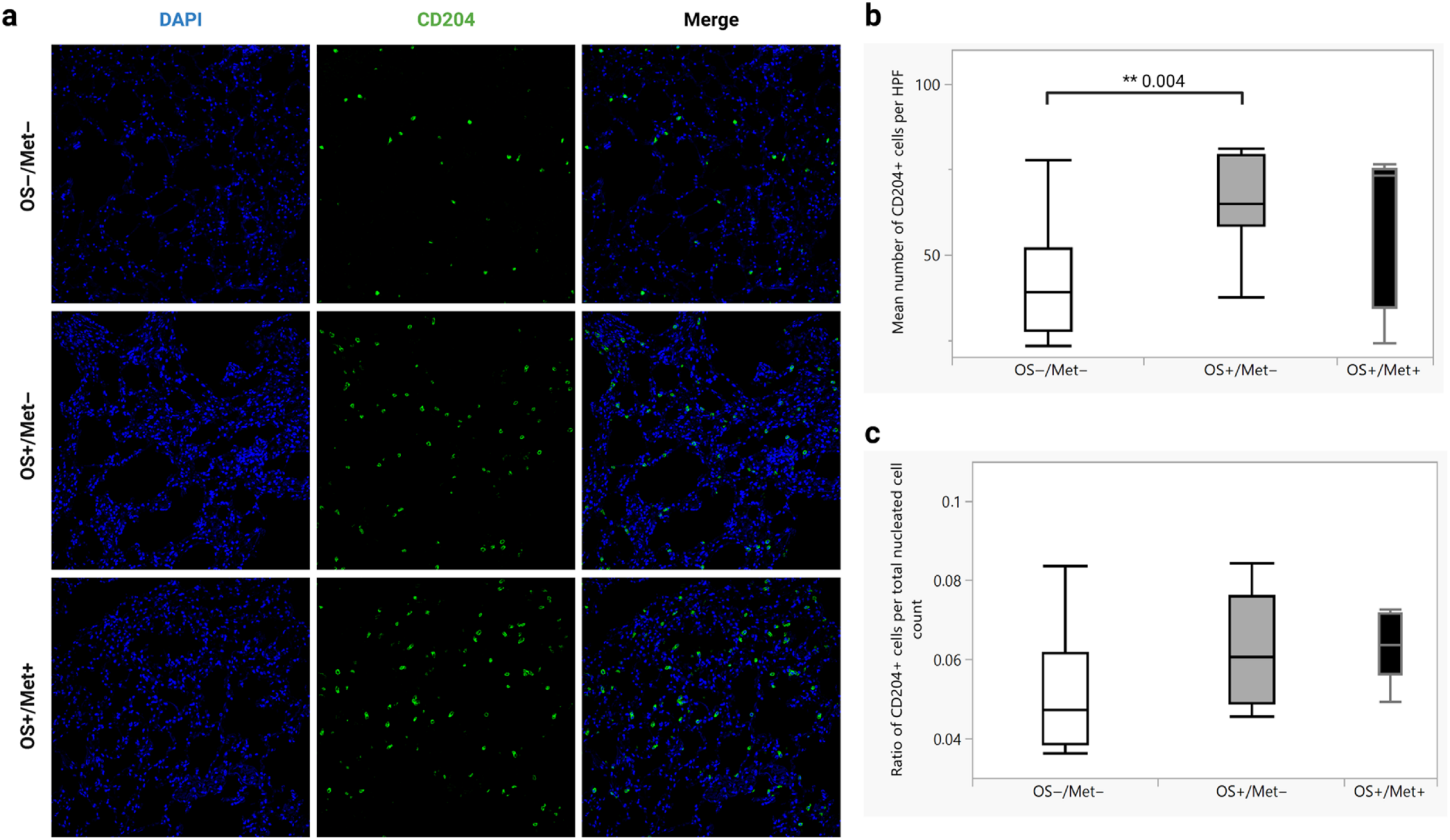
Dogs with osteosarcoma (OS) have more CD204^+^ cells in the lungs before metastasis than control dogs. (a) Immunofluorescent staining for CD204 of formalin‐fixed paraffin‐embedded lung tissue of dogs with OS with metastasis (OS+/Met+), without metastasis (OS+/Met−) and control dogs without cancer (OS−/Met−). Alexa fluor 647 (goat‐anti mouse IgG1) was used as the secondary antibody. Magnification 200×. (b) Quantification of the mean number of CD204^+^ cells per high power field (HPF, 200× magnification, equivalent to 0.276 mm^2^) for each group in (a) (10 randomly selected HPF were counted for each dog). **p < 0.001 calculated using Wilcoxon rank‐sum tests (*n* = 5 in OS+/Met+, *n* = 10 in OS+/Met− and *n* = 10 in OS−/Met−). Box plots show median values and interquartile ranges. (c) Mean ratios of the number of CD204^+^ cells per HPF divided by the total number of nucleated cells in the same HPF for each group in (a). Box plots show median values and interquartile ranges.

The mean number of CD204^+^ macrophages in the lungs in the OS+/Met− group (mean 78.6, CI 46.4–110.7) was significantly higher than in the OS−/Met− group (mean 41.2, CI 28.9–53.5) (*p* = 0.004) (Figure 1b). However, when normalised to the total number of nucleated cells, the means were no longer significantly different (*p* = 0.054) (Figure 1c). The mean number of CD204^+^ cells was not significantly different between the OS+/Met+ and the OS+/Met− (*p* = 0.50) or the OS−/Met− groups (*p* = 0.20), nor when normalised to the total number of nucleated cells (*p* = 0.58 and *p* = 0.16, respectively). Representative images of CD204 immunolabelled lung tissues from each group are shown in Figure 1a.

**FIGURE 2:**
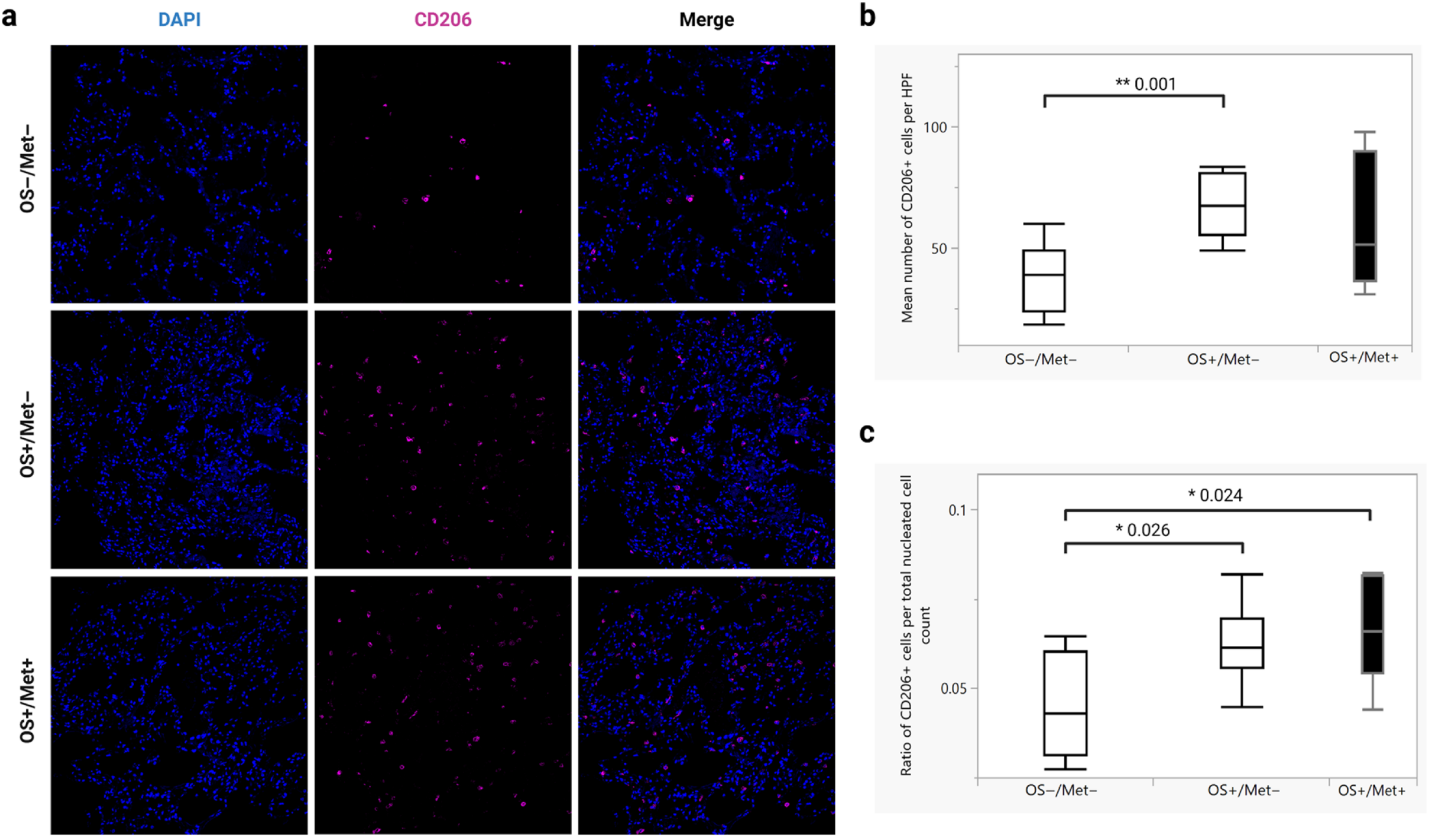
Dogs with osteosarcoma (OS) have more CD206+ cells in the lungs before metastasis than control dogs. (a) Immunofluorescent staining for CD206 of formalin‐fixed paraffin‐embedded lung tissue of dogs with OS with metastasis (OS+/Met+), without metastasis (OS+/Met−) and control dogs without cancer (OS−/Met−). Alexa fluor 750 (goat‐anti rabbit IgG) was used as the secondary antibody. Magnification 200×. (b) Quantification of the mean number of CD206^+^ cells per high power field (HPF, 200× magnification, equivalent to 0.276 mm^2^) for each group in (a) (10 randomly selected HPF were counted for each dog). **p < 0.001 calculated using Wilcoxon rank‐sum tests (*n* = 5 in OS+/Met+, *n* = 10 in OS+/Met− and *n* = 10 in OS−/Met−). Box plots show median values and interquartile ranges. (c) Mean ratios of the number of CD206^+^ cells per HPF divided by the total number of nucleated cells in the same HPF for each group in (a). *p < 0.05 calculated using Wilcoxon rank‐sum tests (*n* = 5 in OS+/Met+, *n* = 10 in OS+/Met− and *n* = 10 in OS−/Met−). Box plots show median values and interquartile ranges.

The mean number of CD206^+^ cells was significantly higher in the OS+/Met− group (mean 84.0, CI 41.3–126.8) than in the OS−/Met− group (mean 38.1, CI 27.2–48.9) (*p* = 0.001) (Figure 2b). This difference remained significant when normalised to the total number of nucleated cells (*p* = 0.026) (Figure 2c). The mean number of CD206^+^ cells was not significantly different between the OS+/Met+ and the OS+/Met− (*p* = 0.50) or the OS−/Met− groups (*p* = 0.14), nor between OS+/Met+ and OS+/Met− when normalised to the total number of nucleated cells (*p* = 0.43). However, the normalised mean number of CD206^+^ cells was significantly higher in the OS+/Met+ group than in the OS−/Met− group (*p* = 0.024) (Figure 2c). Representative images of CD206 immunolabelled lung tissues from each group are shown in Figure 2a.

**FIGURE 3:**
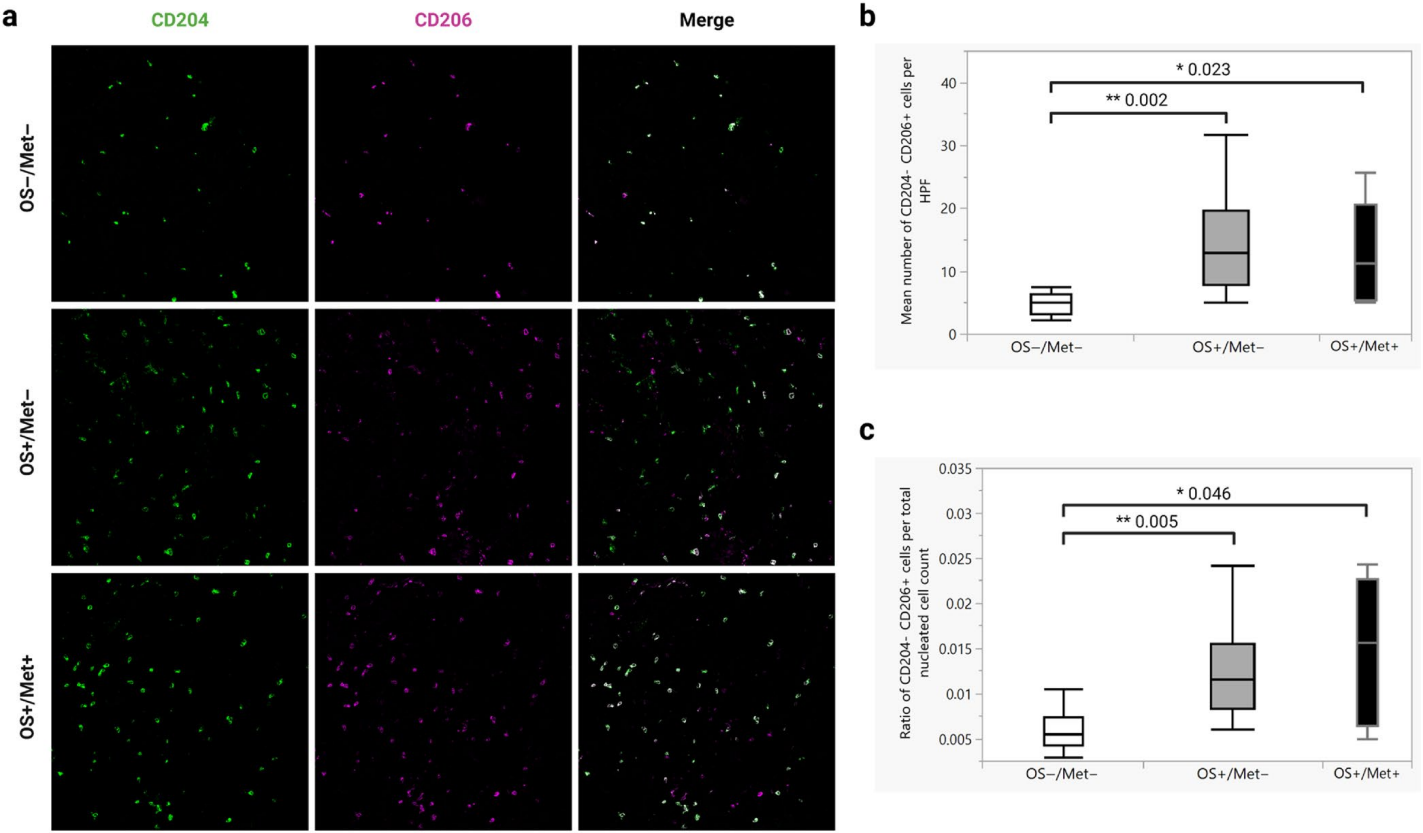
Dogs with osteosarcoma (OS) have more CD204^−^CD206^+^ cells in the lungs before and after metastasis than control dogs. (a) Immunofluorescent staining for CD204 and CD206 of formalin‐fixed paraffin‐embedded lung tissue of dogs with OS with metastasis (OS+/Met+), without metastasis (OS+/Met−) and control dogs without cancer (OS−/Met−). Alexa fluor 647 (goat‐anti mouse IgG1) was used as the secondary antibody for CD204 and Alexa fluor 750 (goat‐anti rabbit IgG) for CD206. Magnification 200×. (b) Quantification of the mean number of CD204^−^CD206^+^ cells per high power field (HPF, 200× magnification, equivalent to 0.276 mm^2^) for each group in (a) (10 randomly selected HPF were counted for each dog). *p < 0.05 and **p < 0.001 calculated using Wilcoxon rank‐sum tests (*n* = 5 in OS+/Met+, *n* = 10 in OS+/Met− and *n* = 10 in OS−/Met−). Box plots show median values and interquartile ranges. (c) Mean ratios of the number of CD204^−^CD206^+^ cells per HPF divided by the total number of nucleated cells in the same HPF for each group in (a). *p < 0.05 and **p < 0.001 calculated using Wilcoxon rank‐sum tests (*n* = 5 in OS+/Met+, *n* = 10 in OS+/Met− and *n* = 10 in OS−/Met−). Box plots show median values and interquartile ranges.

A subpopulation of CD206^+^ cells did not express CD204. These CD204^−^CD206^+^ cells' morphology was consistent with interstitial macrophages or monocytes. All alveolar macrophages were CD204^+^. The mean number of CD204^−^CD206^+^ cells was significantly higher in the OS+/Met− group (mean 14.2, CI 8.6–19.9) than in the OS−/Met− group (mean 4.7, CI 3.4–6.1) (*p* = 0.002) (Figure 3b). This difference remained significant after normalising to the total number of nucleated cells (*p* = 0.005) (Figure 3c). The mean number of CD204^−^CD206^+^ cells was not significantly different between the OS+/Met+ and the OS+/Met− groups (*p* = 0.76), nor when normalised to the total number of nucleated cells (*p* = 0.67). However, the mean number of CD204^−^CD206^+^ cells was significantly higher in the OS+/Met+ group than in the OS−/Met− group (*p* = 0.023) (Figure 3b), also when normalised to the total number of nucleated cells (*p* = 0.046) (Figure 3c). Representative images of double immunolabelled (CD204 and CD206) lung tissues from each group are shown in Figure 3a.

### 3.3 Cases With OS Have Significantly Higher Numbers of CD11d ^+^ Bone Marrow‐Derived Cells in the Lungs Before Metastasis Than Those With Metastases and Controls

**FIGURE 4:**
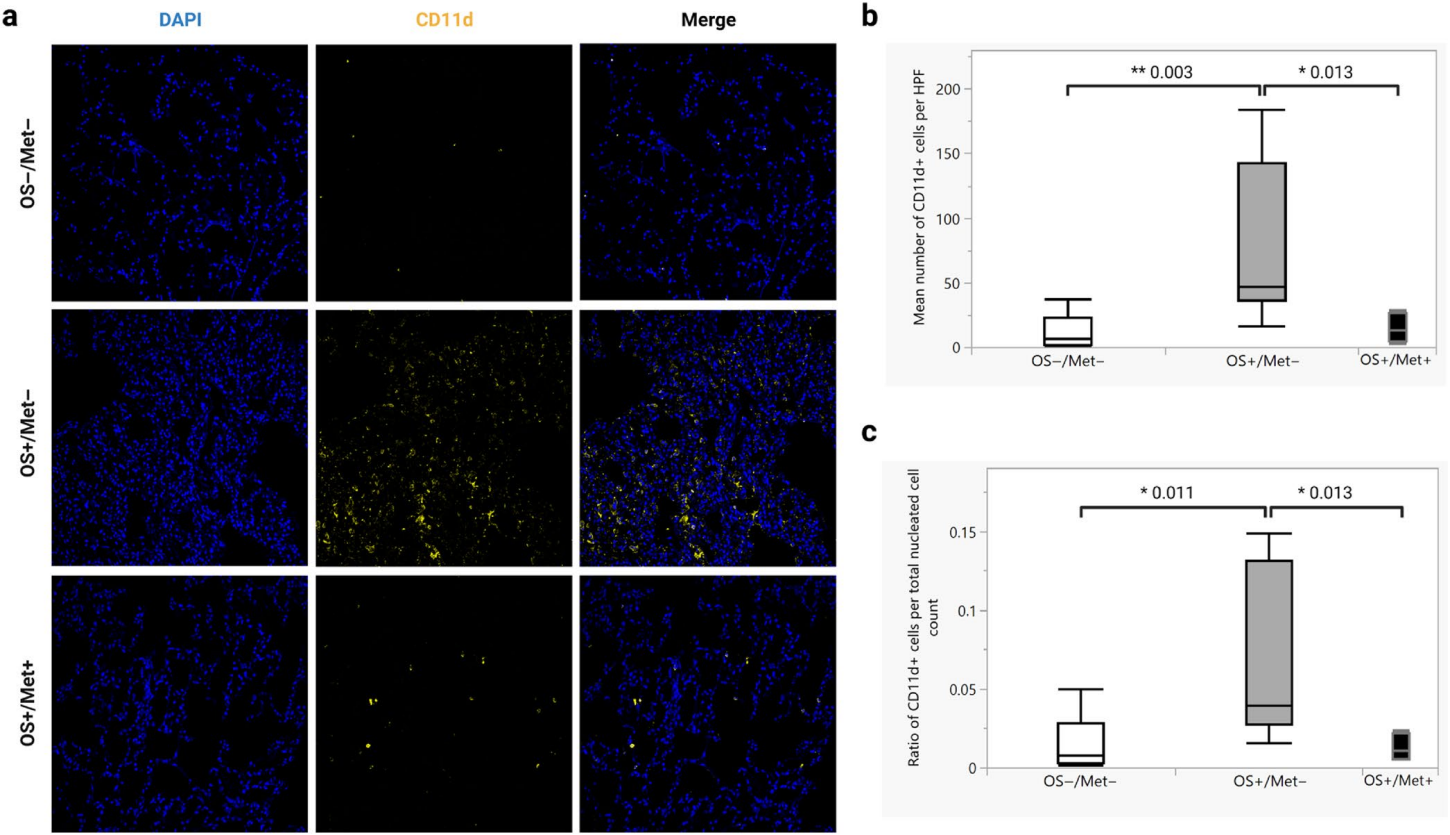
Dogs with osteosarcoma (OS) have more CD11d^+^ cells in the lungs before metastasis than those with established metastases and control dogs. (a) Immunofluorescent staining for CD11d of formalin‐fixed paraffin‐embedded lung tissue of dogs with OS with metastasis (OS+/Met+), without metastasis (OS+/Met−) and control dogs without cancer (OS−/Met−). Alexa fluor 647 (goat‐anti mouse IgG1) was used as the secondary antibody. Magnification 200×. (b) Quantification of the mean number of CD11d^+^ cells per high power field (HPF, 200× magnification, equivalent to 0.276 mm^2^) for each group in (a) (10 randomly selected HPF were counted for each dog). *p < 0.05 and **p < 0.001 calculated using Wilcoxon rank‐sum tests (*n* = 5 in OS+/Met+, *n* = 10 in OS+/Met− and *n* = 10 in OS−/Met−). Box plots show median values and interquartile ranges. (c) Mean ratios of the number of CD11d^+^ cells per HPF divided by the total number of nucleated cells in the same HPF for each group in (a). *p < 0.05 calculated using Wilcoxon rank‐sum tests (*n* = 5 in OS+/Met+, *n* = 10 in OS+/Met− and *n* = 10 in OS−/Met−). Box plots show median values and interquartile ranges.

The mean number of CD11d^+^ myeloid cells in the lungs in the OS+/Met− group (mean 77.5, CI 34.7–120.3) was significantly higher than in the OS−/Met− group (mean 19.5, CI 0.0–41.5) (*p* = 0.003) (Figure 4b). This was also the case when normalised to the total number of nucleated cells (*p* = 0.011) (Figure 4c). The mean number of CD11d^+^ cells was also significantly higher in the OS+/Met− group than in the OS+/Met+ (mean 14.8, CI 0.0–33.0) (*p* = 0.013) (Figure 4b), also when normalised to the total number of nucleated cells (*p* = 0.013) (Figure 4c). The number of CD11d^+^ cells was not significantly different between the OS+/Met+ group and the OS−/Met− group (*p* = 0.52), nor when normalised to the total number of nucleated cells (*p* = 0.94). CD11d^+^ cells were almost exclusively CD206^−^. Representative images of CD11d immunolabelled lung tissues from each group are shown in Figure 4a.

### 3.4 The Ratio of CD206 ^+^ CD204 ^+^/CD204 ^+^ Macrophages in the Lungs Remains Unchanged in OS Cases, Compared to Controls

The ratio of CD206^+^CD204^+^/CD204^+^ cells was similar among all three groups. The ratio in the OS+/Met− group was 0.83 (CI 0.77–0.89), which was not significantly different from the OS−/Met− group (0.76, CI 0.72–0.84) (*p* = 0.18) or the OS+/Met+ group (0.81, CI 0.66–0.96) (*p* = 0.85), nor was there any difference between the OS−/Met− and OS+/Met+ groups (*p* = 0.42). These CD206^+^CD204^+^double‐positive cells represented alveolar and interstitial macrophages.

Some CD204^+^ cells were CD206^−^, but there was no significant difference in the mean number between the OS+/Met− and OS−/Met− (*p* = 0.24) or the OS+/Met+ groups (*p* = 0.50), nor between the OS+/Met+ and OS−/Met− groups (*p* = 0.89). This was the same when normalised to the total number of nucleated cells (*p* = 0.71, *p* = 0.89 and *p* = 0.95, respectively).

### 3.5 The Total Nucleated Cell Density of the Lung Tissue Is Significantly Higher in OS Cases Before Metastasis Than in Controls

The mean number of total nucleated cells (DAPI^+^ cells) per HPF was significantly higher in the OS+/Met− group (1106.0, CI 949.1–1263.0) than in the OS−/Met− group (778.1, CI 682.6–873.6) (*p* = 0.004) (Figure S5). There was, however, no significant difference in the mean number of total nucleated cells between the OS+/Met+ group and the OS+/Met− group (*p* = 0.27) or the OS−/Met− group (*p* = 0.50).

## 4 Discussion

Despite many reports of PMN formation in distant metastatic target organs in murine models, none have reported evidence of its existence in naturally occurring cancer. Microenvironmental changes compatible with PMN formation have been described in tumour‐draining lymph nodes of patients with malignant cancers such as melanoma, cervical cancer, breast cancer, lung cancer and oral squamous carcinomas [18, 19, 20, 21, 22]. In this study, we have found evidence supporting the existence of a pulmonary PMN in dogs with naturally occurring OS. Dogs with OS had significantly higher numbers of BMDCs, macrophages and monocytes in the pre‐metastatic lungs than dogs without cancer.

One of the main challenges when researching PMN in humans is accessing tissues from metastatic target organs before metastasis occurs. Furthermore, most tissues from human cancer patients have been exposed to treatments that could interfere with the niche. Our findings are similar to those reported in murine xenograft OS models of PMN [23]. Dogs with cancer may therefore represent a unique opportunity, as many owners elect euthanasia over treatment due to financial or ethical reasons or a lack of standardised and effective treatment options. Therefore, many dogs do not receive treatment for their cancer, which could affect the PMN.

Previous studies of pulmonary PMN formation have shown that several types of tumours can induce the recruitment of different immune cells to the lungs before metastasis [4, 6]. One of the most striking changes reported is the recruitment of CD11b^+^ BMDCs [4, 6]. In murine xenograft models of OS, most CD11b^+^ cells also express Gr‐1, suggesting they are either neutrophils or granulocytic MDSC [23]. Additionally, MDSC, VLA‐4^+^ BMDCs, VEGFR1^+^ BMDCs, macrophages, monocytes and Tregs are recruited to the lungs before metastasis [4, 6]. We found an increased number of CD11d^+^ cells in the pre‐metastatic lung of dogs with OS, which are usually not present in healthy canine lungs [24]. CD11d^+^ is a β2‐integrin first described in dogs and expressed by a subset of canine bone marrow macrophages, splenic red pulp macrophages and lymph node medullary macrophages, as well as a small subset of circulating CD8^+^ lymphocytes [24]. Canine granulocytic and dendritic cells do not express CD11d, suggesting these positive cells are not granulocytes but rather monocytic BMDCs. It is also possible that a subset of these CD11d^+^ cells represents monocytic MDSC. However, CD11d expression in canine MDSC has not been investigated [25]. In humans, most myeloid cells express CD11d, including circulating monocytes, monocyte‐derived macrophages, macrophages, dendritic cells and neutrophilic granulocytes [26]. In addition, leukocytes in the alveoli and alveolar septa express CD11d under physiologic conditions in humans. Therefore, CD11d would not serve as a good marker for BMDC recruitment to the PMN in humans. The CD11d^+^ BMDCs we observed might also have been CD11b^+^, but this epitope is destroyed by formalin fixation in canine tissues and could not be assessed.

We also found an increased number of CD204^+^ macrophages in the pre‐metastatic lung of dogs with OS. This is in line with findings from murine models of pancreatic cancer and salivary cystic carcinoma, where tumour‐derived exosomes resulted in increased numbers of F4/80^+^ macrophages in the liver and the lungs, respectively [27, 28]. CD204 is a scavenger receptor expressed by tissue‐resident and monocyte‐derived macrophages in dogs [29]. Alveolar and interstitial macrophages express CD204 under physiologic conditions [29]. The increased number of CD204^+^ cells reflects an increased total number of macrophages. Whether this increase is due to the local expansion of tissue‐resident macrophages or recruited monocyte‐derived macrophages is unclear.

The mannose receptor CD206 is a useful M2 macrophage phenotypical marker in humans and mice [30, 31]. It has been well established that tumour‐associated macrophages (TAMs) have a predominantly M2‐skewed tumour‐permissive phenotype, with high CD206 expression [32]. Similarly, CD206 is highly expressed in canine M2 macrophages, and most TAMs in dogs with malignant mammary tumours express CD206 [33, 34]. We found an increased number of CD206^+^ macrophages in the lungs of dogs with OS, suggesting there is an increase in M2‐skewed immunosuppressive and tumour‐permissive macrophages before and after metastasis. Furthermore, dogs with OS had a higher number of CD204^−^CD206^+^ cells, with morphology compatible with undifferentiated monocytes. This suggests that the increased numbers are partly explained by monocyte recruitment and not due to the expansion of tissue‐resident macrophages alone. There was no significant difference in the ratio of CD206^+^CD204^+^/CD204^+^ cells between groups. As in humans, most alveolar and interstitial macrophages in healthy lungs express CD206 and are considered M2‐skewed under physiologic conditions [35]. Furthermore, almost all TAMs in pulmonary metastases were CD206^+^ (Figure S6).

The numbers of positive cells were normalised against the total number of nucleated cells to account for differences in tissue density between dogs. However, we also found that dogs with OS had significantly higher numbers of nucleated cells per HPF in the pre‐metastatic lung than controls. There was no significant difference in nucleated cell density between dogs with OS with and without metastases. However, this may be due to the low number of dogs in the OS+/Met+ group. The cell density varied more between the groups than between individual dogs. This increase in tissue density was of such a magnitude that it could not only be attributed to the increased numbers of CD11d^+^ BMDCs, macrophages and monocytes alone. Other leukocyte populations, such as granulocytes and Tregs, or stromal cell populations, such as fibroblasts, could explain the difference.

Interestingly, we found similar numbers of CD206^+^ and CD204^+^ cells in dogs with OS with and without established metastases. Unfortunately, there were few dogs in the OS+/Met+ group, and therefore, the study could have been too underpowered to detect any such difference. However, the number of CD11d^+^ BMDCs was significantly higher among the dogs without metastases than those with established metastases. These results suggest that the recruitment of CD11d^+^ cells might be a transient event necessary for metastatic development and ceases once metastases have developed.

Although we did not explore NK cell dysfunction and hampered cytotoxic CD8^+^ T‐cell responses, M2‐skewed macrophages and tumour‐conditioned macrophages are known to interfere with these responses [36, 37, 38]. Similarly, MDSCs in the PMN in mice have been shown to inhibit anti‐tumour T‐cell responses by expressing Th2 cytokines and reducing IFN‐γ production [39]. In addition, MDCSs can inhibit NK‐cell mediated cytotoxicity through TGF‐β, antigen‐presentation by dendritic cells and macrophages through IL‐10 and convert effector T‐cells into Tregs [40, 41, 42]. Although not confirmed, it is probable that a subset of the observed CD11d^+^ cells represent MDSC. Many of the CD11b^+^ cells in the murine PMN also express MDSC markers such as Ly6G and Ly6C [6]. Since angiogenesis and vascular leakiness are challenging to study in a clinical setting in dogs, we could not explore these aspects of PMN formation. However, M2‐skewed macrophages and TAMs secrete vascular endothelial growth factors and matrix metalloproteinase (MMPs) 1, 9 and 12, which can induce angiogenesis and increase vascular permeability [6, 43].

When examining canine patients with naturally occurring cancer, there is more variation between cases than when performing controlled pre‐clinical laboratory experiments. There is inter‐tumour variation, host differences such as breed, age and weight and environmental and dietary differences. Age can significantly influence the competence of the immune response in dogs, as well as immune cell subset composition [44]. However, there were no significant differences in age between the groups in our study. Lymphocyte subsets compositions can vary between breeds [45]. However, differences in tissue‐resident and infiltrating immune cell subsets in the between breeds remains poorly characterised. The dogs in the OS−/Met− group were significantly smaller than the dogs with OS, mainly due to differences in breed composition. Therefore, whether the observed differences in myeloid infiltrates observed between the groups in our study could have been influenced by breed composition is therefore unclear. Furthermore, previous diseases or medications could have been missed during retrospective data collection or due to recollection bias in owners when recording the medical history. In addition, the dogs were necropsied and sampled at different time points in their disease progression. Although most dogs (> 90%) develop pulmonary metastases after complete surgical removal of the primary tumour, there is no guarantee that all the included dogs without visible metastases would have developed them [14, 15, 16]. The fact that there were significant differences in a relatively small material supports the concept that PMN formation is a common phenomenon in OS. Lastly, there might have been a difference in the time between euthanasia and necropsy between the prospectively and retrospectively collected dogs. Prospectively collected dogs with OS were probably necropsied within a shorter time from euthanasia, compared to the retrospectively collected cases. Whether a prolonged time from death to euthanasia results in removal of myeloid cells either by cell migration away from the lung tissues or necrosis is unclear, however, considered unlikely by the authors.

Changes compatible with PMN formation usually only occur in organs that subsequently develop metastases in murine models [3, 6]. Although we found changes compatible with PMN formation in the lungs of dogs with OS before metastases, we did not assess any other non‐metastatic target organs. It is therefore possible that the described changes may be present in other organs too and represent a systemic reaction.

Results from pre‐clinical research on new cancer drugs in murine models show a poor correlation with later clinical efficacy in human cancer patients [46]. Evidence of PMN formation in dogs with OS supports their use as models to assess PMN‐targeting drugs. The macrophage‐repolarizing drug muramyl tripeptide (MTP) significantly improved survival in dogs with OS after surgical removal of the primary tumour (median survival time of 222 days vs. 77) [47]. MTP can repolarize alveolar macrophages from an M2‐skewed phenotype towards an M1 phenotype in vitro [48]. These dogs received MTP at a time point in their disease process where only microscopic disease was present. A potential explanation for the clinical benefit could be MTP's ability to reprogram the immunosuppressive pulmonary PMN. MTP was also later found to improve survival in human paediatric patients with OS [49].

In conclusion, we found evidence supporting the existence of a PMN in an organism with naturally occurring cancer. Further studies investigating other aspects of PMN formation in dogs with OS and other highly metastatic cancers are therefore warranted. Dogs with OS may represent excellent candidates for assessing microenvironmental changes in metastatic target tissues and new PMN‐targeting drugs.

## Author Contributions

M.K., F.L., B.K.S. and L.M. participated in the collection of material. J.T. performed the histological examination of all tissue samples. M.K. performed the immunofluorescent staining, image acquisition and analysis. M.K., K.P.A., E.O.K., F.L., D.A. and L.M. participated in the interpretation of data. M.K., K.P.A., E.O.K., F.L., D.A., J.T., B.K.S. and L.M. contributed to writing the manuscript. All authors have read and approved the final version of the manuscript.

## Conflicts of Interest

The authors declare no conflicts of interest.

## Supporting information


Data S1.

## Data Availability

The data that support the findings of this study are available from the corresponding author upon reasonable request. No cell lines were used in this study.
