# Supplementary material for: Immunological Pre‐Metastatic Niche in Dogs With Naturally Occurring Osteosarcoma
Source: Vet Comp Oncol. 2024 Nov 11;23(1):62–72. doi: 10.1111/vco.13026 (PMC11830463; doi:10.1111/vco.13026)
Supplement: Supplementary file 1 — Data S1. [file VCO-23-62-s001.docx]

**Supplementary files**

**Immunological pre-metastatic niche in dogs with naturally occurring osteosarcoma**

**This PDF file includes:**

Supplementary Figure 1-6


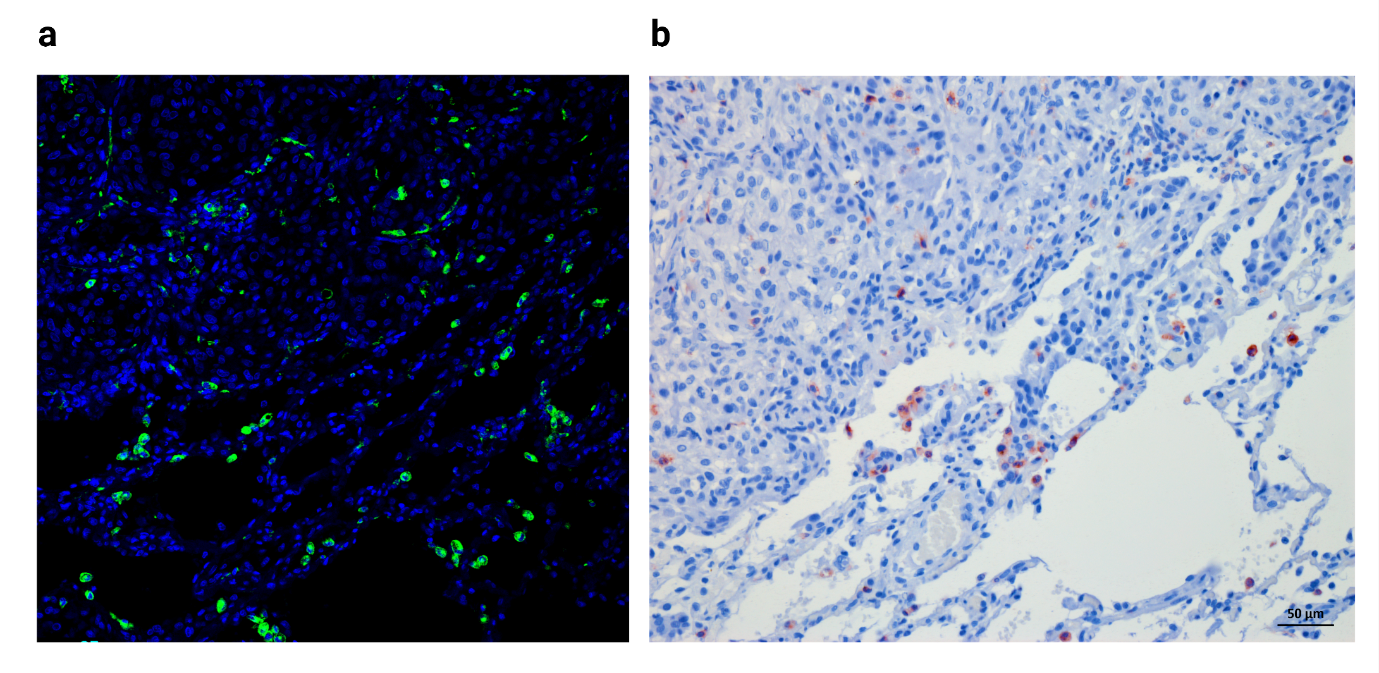


**Supplementary Fig. 1** Comparison of immunofluorescence (IF) and chromogen immunohistochemical (IHC) staining for CD204. **a** IF staining for CD204 of formalin-fixed paraffin-embedded lung tissue of a dog with osteosarcoma with metastases. Alexa fluor 647 (goat-anti mouse IgG1) was used as the secondary antibody. Magnification 200x. **b** IHC staining for CD204 of the same tissue block. 3-amino-9-ethyl carbazole (AEC) chromogen and Meyer’s hematoxylin counterstain. Magnification 200x. Scale bar: 50 µm.

**
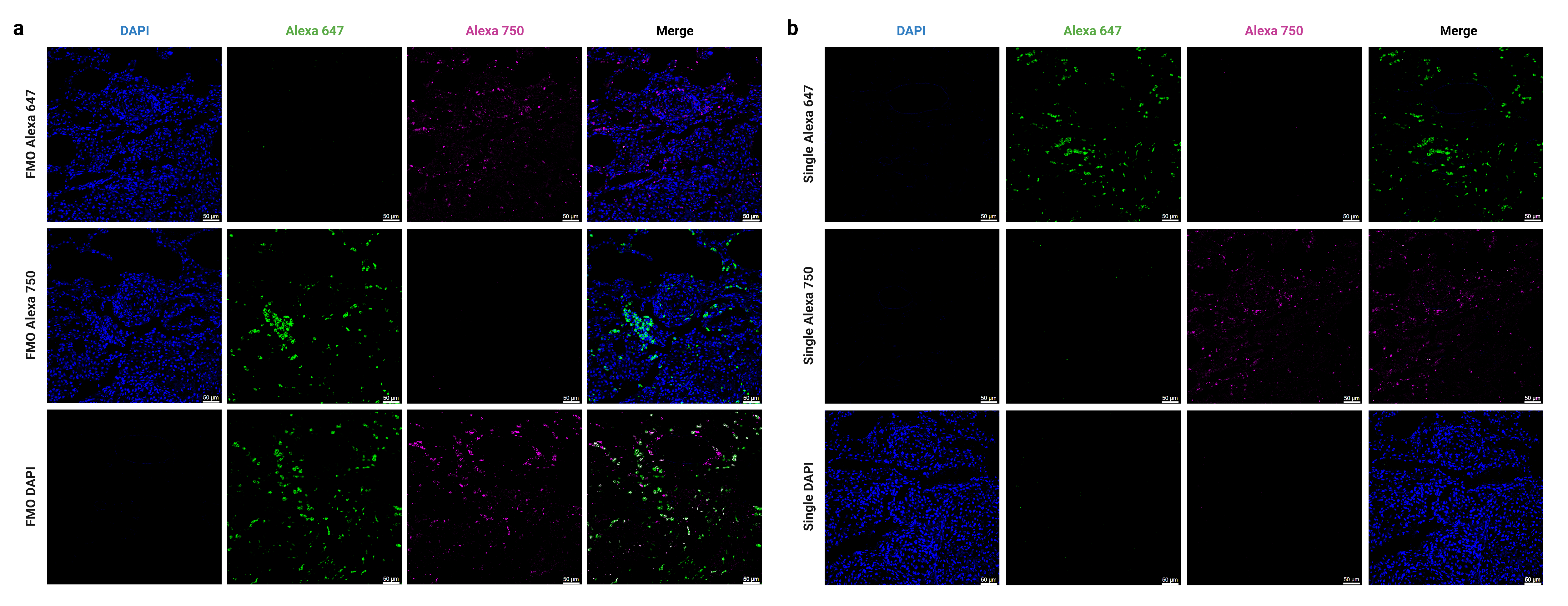
**

**Supplementary Fig. 2** Fluorescence minus one (FMO) controls and single controls for the CD204/CD206 antibody panel. **a** FMO controls for DAPI, Alexa fluor 647 (primary antibody mouse anti-CD204), and Alexa Fluor 750 (primary antibody rabbit anti-CD206). In each row, one of the primary antibodies or DAPI has been omitted, while all secondary antibodies have been included. There is no spectral overlap between the channels or unspecific binding of secondary antibodies. **b** Single control for DAPI, Alexa fluor 647 (primary antibody anti-CD204), and Alexa Fluor 750 (primary antibody anti-CD206). In each row, only one of the primary antibodies or DAPI was used, while all secondary antibodies were included. There is no bleed-thru of signal between channels or unspecific binding of secondary antibodies to the primary antibodies. Magnification: 200x. Scale bar: 50 µm.


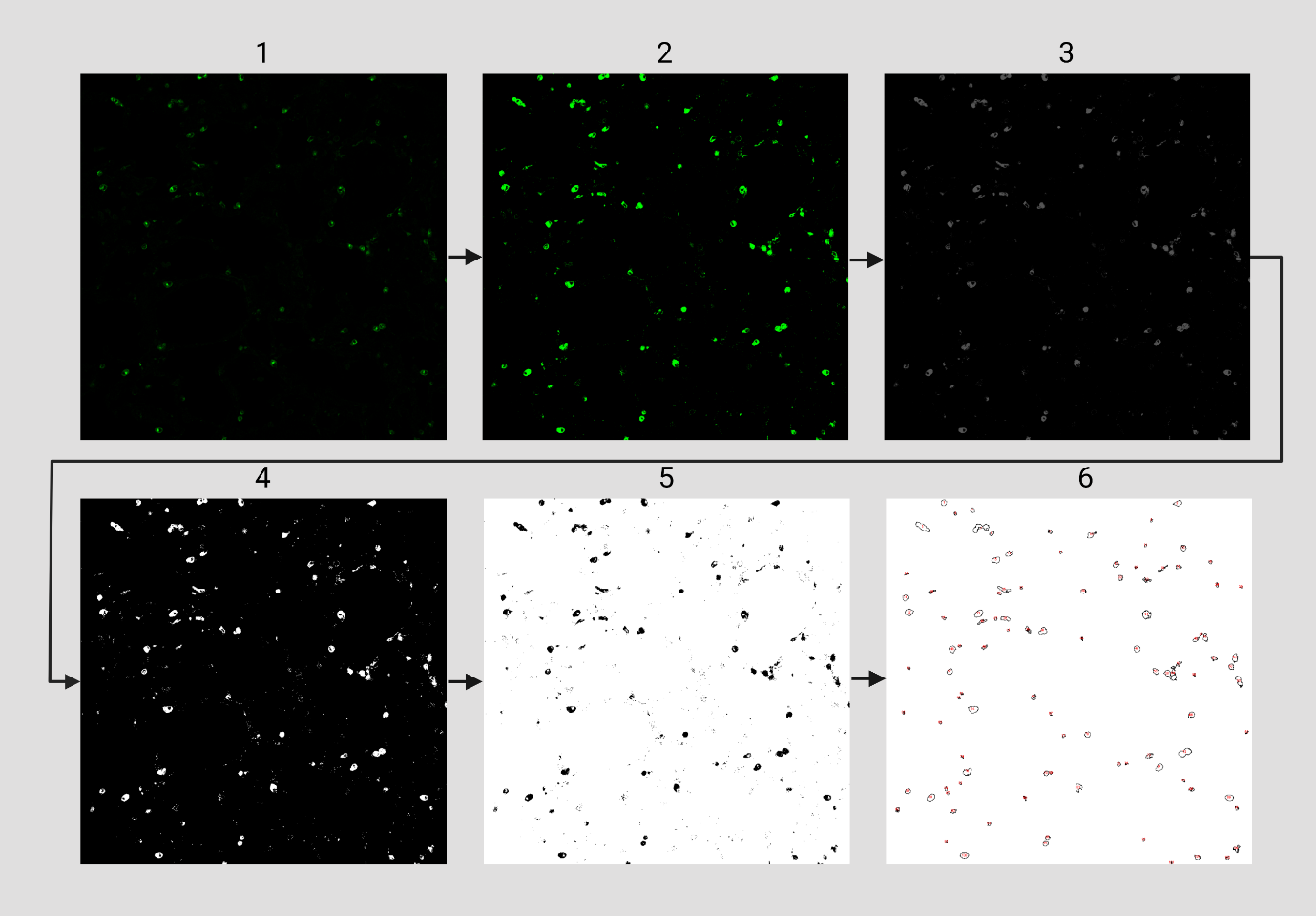


**Supplementary Fig. 3** Visualization of automated positive cell quantification using ImageJ. **1** Images were opened in ImageJ and **2** image brightness was adjusted. **3** Images were then then converted to 8-bit grayscale, before **4** threshold was adjusted to remove background noise. **5** Images were then inverted, and holes filled, before **6** the number of positive cells were counted using the particle analysis tool counting particles with a size larger than 20 pixel units.


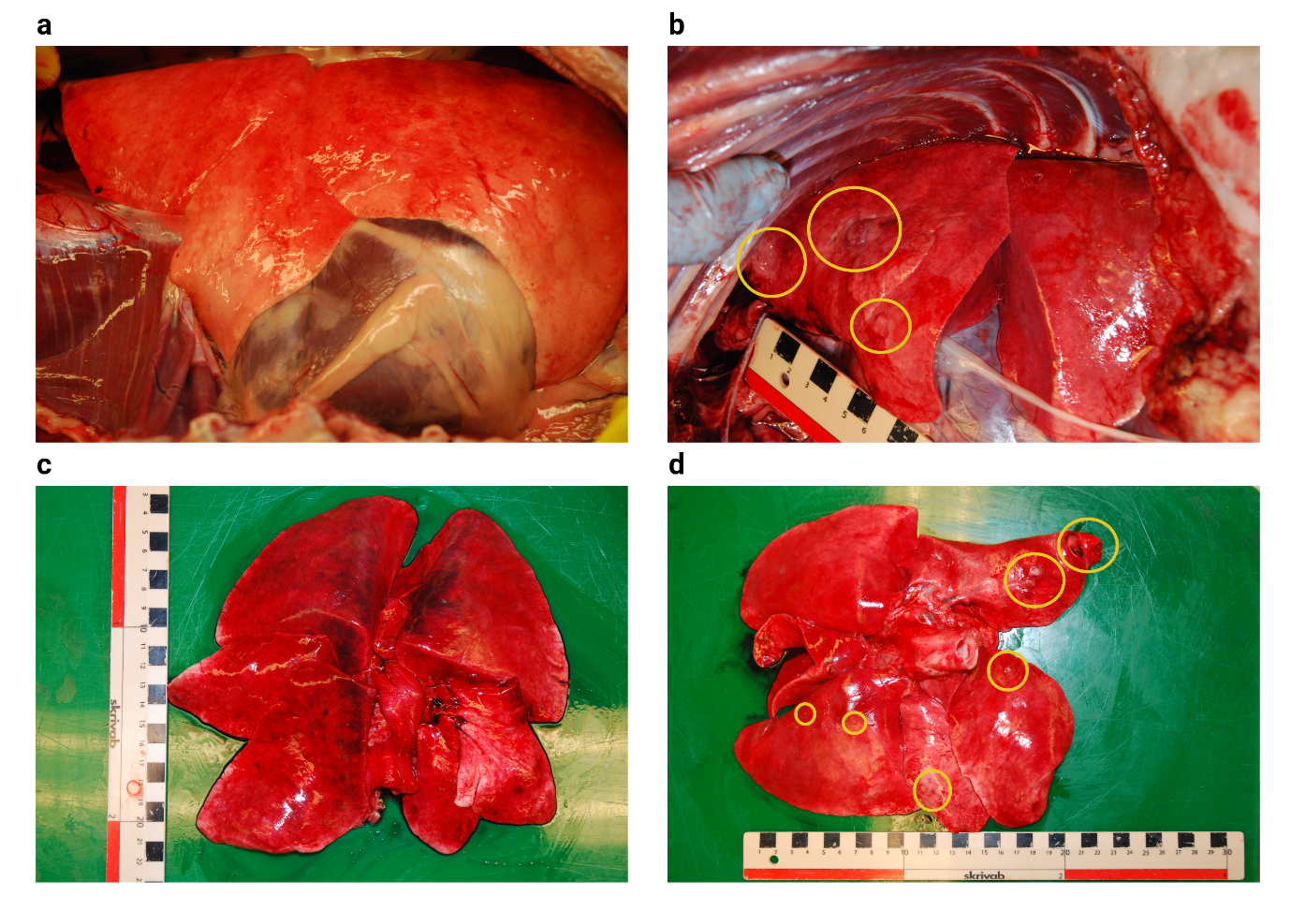


**Supplementary Fig. 4** Macroscopic pictures of the lungs of dogs with osteosarcoma (OS) with and without metastasis. **a** Picture of the lung *in situ* in the right thoracic cavity of a dog with OS without pulmonary metastases. **b** Picture of the lung *in situ* in the left thoracic cavity of a dog with OS with pulmonary metastases. **c** Picture of the same lungs as in **a** after removal from the dog. **d** Picture of the same lungs as in **d** after removal from the dog. Metastases are indicated with yellow circles.


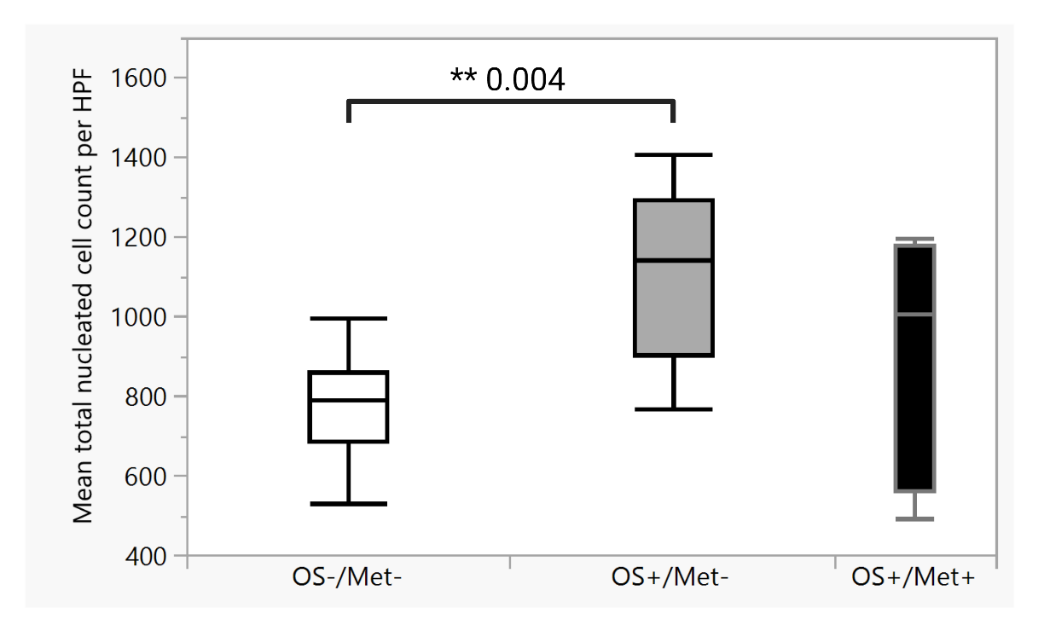


**Supplementary Fig. 5** Dogs with osteosarcoma (OS) have a higher total nucleated cell density in the pre-metastatic lung than controls. Quantification of the mean number of DAPI^+^ cells per high power field (HPF, 200x magnification, equivalent to 0.276mm^2^) for each group (10 randomly selected HPF were counted for each dog). ^**^P < 0.001 calculated using Wilcoxon rank-sum tests (n= 5 in OS+/Met+, n=10 in OS+/Met-, and n=10 in OS-/Met-). Box plots show median values and interquartile ranges.


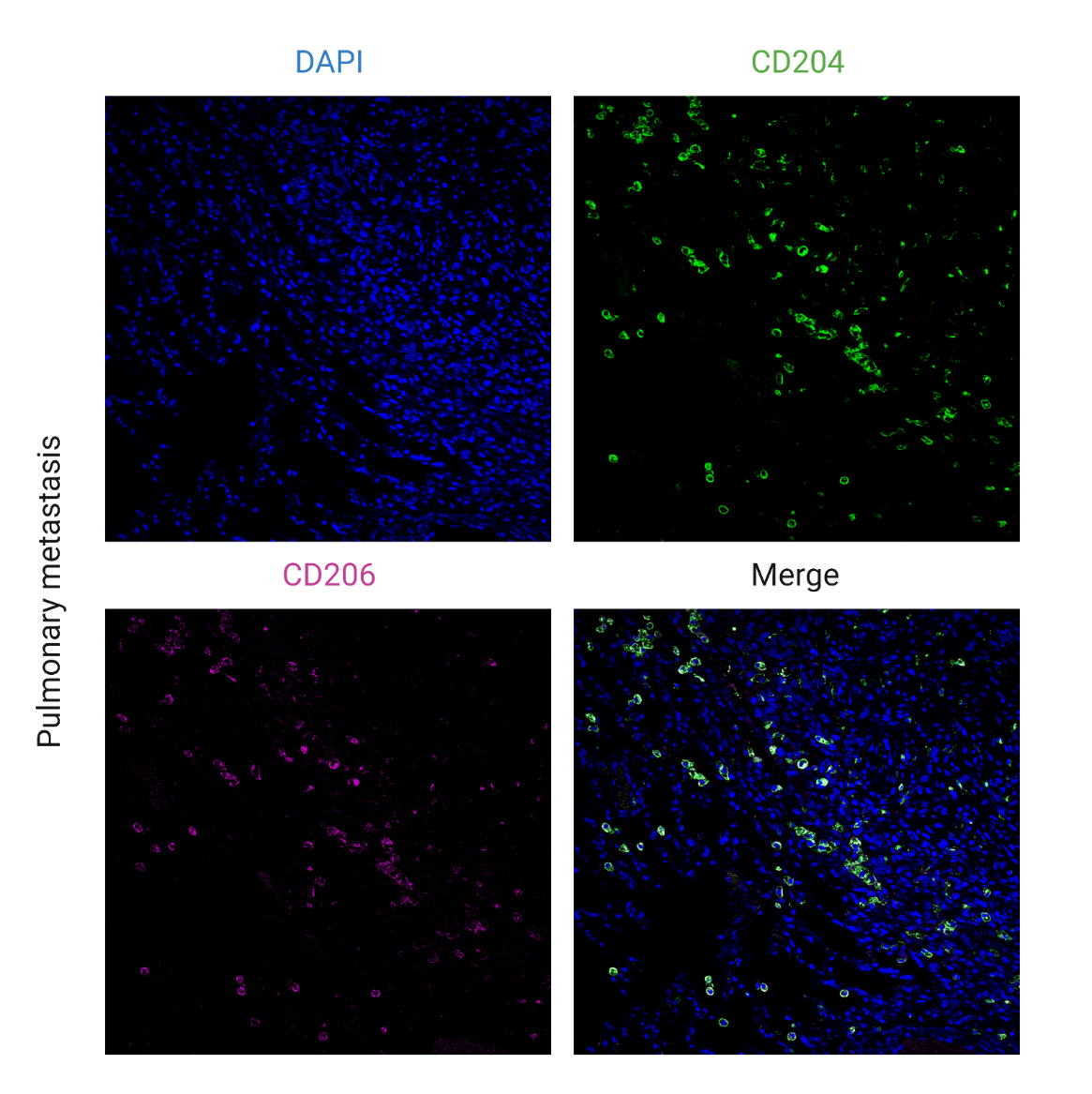


**Supplementary Fig. 6** Tumor-associated macrophages (TAMs) within pulmonary metastases of osteosarcoma (OS) express the M2-associated marker CD206. Immunofluorescent staining for CD204, CD206, and DAPI of formalin-fixed paraffin-embedded lung tissue from a dog with OS with metastasis. Alexa fluor 647 (goat-anti mouse IgG1) was used as the secondary antibody for CD204 and Alexa fluor 750 (goat-anti rabbit IgG) for CD206. Magnification 200x. Virtually all TAMs express both CD204 and CD206.
